# Early Childhood Pneumonia Is Associated with Reduced Lung Function and Asthma in First Nations Australian Children and Young Adults

**DOI:** 10.3390/jcm10245727

**Published:** 2021-12-07

**Authors:** Andrew J. Collaro, Anne B. Chang, Julie M. Marchant, Mark D. Chatfield, Don Vicendese, Tamara L. Blake, Margaret S. McElrea, Shyamali C. Dharmage

**Affiliations:** 1 Department of Respiratory and Sleep Medicine, Queensland Children’s Hospital, Brisbane, QLD 4101, Australia; anne.chang@menzies.edu.au (A.B.C.); jm.marchant@qut.edu.au (J.M.M.); t.blake@uq.edu.au (T.L.B.); margaret.mcelrea@qut.edu.au (M.S.M.); 2 Australian Centre for Health Services Innovation, Queensland University of Technology, Brisbane, QLD 4059, Australia; 3 Child Health Division, Menzies School of Health Research, Darwin, NT 0811, Australia; m.chatfield@uq.edu.au; 4 Faculty of Medicine, The University of Queensland, Brisbane, QLD 4072, Australia; 5 Allergy and Lung Health Unit, Melbourne School of Population and Global Health, The University of Melbourne, Melbourne, VIC 3053, Australia; don.vicendese@unimelb.edu.au (D.V.); s.dharmage@unimelb.edu.au (S.C.D.)

**Keywords:** pneumonia, early childhood, respiratory tract infection, lrti, spirometry

## Abstract

Background: Some but not all previous studies report that pneumonia in children aged less than five years is associated with lower lung function and elevated risk of respiratory disease. To date, none have explored these associations in at-risk populations such as First Nations Australians, whose incidence of early childhood pneumonia is among the highest reported in the world. Methods: This cross-sectional study included 1276 First Nations Australian children/young adults aged 5–25 years recruited from regional/remote Queensland and Northern Territory communities and schools. Associations between pneumonia and both spirometry values and asthma were investigated using linear and logistic regression. Results: Early childhood pneumonia was associated with lower FEV1 and FVC Z-scores, but not FEV1/FVC% Z-scores, when occurring before age three (FEV1 β = −0.42, [95%CI −0.79, −0.04]; FVC β = −0.62, [95%CI −1.14, −0.09]), and between three and five years (β = −0.50, [95%CI −0.88, −0.12]; β = −0.63, [95%CI −1.17, −0.10]), compared to those who never had pneumonia. Similarly, pneumonia occurring when aged before age three years (OR = 3.68, 95%CI 1.96–6.93) and three to five years (OR = 4.81, 95%CI 1.46–15.8) was associated with increased risk of asthma in later childhood. Conclusions: Early childhood pneumonia is associated with lung function deficits and increased asthma risk in later childhood/early adulthood in First Nations Australians. The disproportionate impact of pneumonia on at-risk children must be addressed as a priority.

## 1. Introduction

Pneumonia is an important global public health issue as it remains the leading cause of morbidity and mortality in children aged under five years, [1,2] with an estimated 156 million cases [2] and nearly one million deaths annually [3]. It disproportionately affects Indigenous children worldwide, including Aboriginal and Torres Strait Islander Australian (henceforth respectfully referred to as First Nations Australian) children, for whom it is the leading cause of preventable death and hospitalisation in those aged less than five years [4]. The potential adverse consequences of this high burden have not yet been investigated in these populations despite increasing, although still inconsistent, evidence of the impact of childhood pneumonia on subsequent lung function and respiratory morbidity.

Childhood pneumonia has been associated with reduced lung function (defined by reduced forced expiratory volume in one second (FEV_1_) and forced vital capacity (FVC) as measured by spirometry) both in later childhood, [5,6,7,8,9,10] where primarily obstructive deficits are seen, and adulthood where restrictive deficits are most common [5,8,11,12,13]. These adverse consequences are greater when pneumonia occurs before five years of age [5,10]. These observations are plausible given that continued airway growth and rapid proliferation of alveoli occur up to three to four years postnatally, and infection in these early years may impair overall lung growth [14]. Low childhood lung function predicts low adulthood lung function [15,16,17,18] which is associated with future cardiopulmonary and all-cause morbidity and mortality, [19] including for First Nations Australians [20].

Despite the increasing evidence on adverse lung function outcomes of childhood pneumonia, there are no such data in populations with reported high risk of severe chronic respiratory disease such as First Nations Australians. The incidence of early childhood respiratory infections in First Nations Australians is among the highest reported in the world [4]. To address these knowledge gaps, we utilised Indigenous Respiratory Reference Values (IRRV) study [21] data to evaluate the effects of documented childhood pneumonia occurring at or before five years of age on lung function in later childhood/early adulthood in 909 First Nations Australian children. We hypothesised that early childhood pneumonia is associated with poorer lung function and asthma, and that the age of pneumonia first occurring modifies these associations, with decreasing age of pneumonia having greater impact.

## 2. Materials and Methods

### 2.1. Study Design, Setting, and Participants

We used cross-sectional data of First Nations Australian children and young adults aged 5–25 years from the Indigenous Respiratory Reference Values (IRRV) study [21]. Participants were approached and recruited opportunistically from childcare centres, schools, and community events from nine Queensland and Northern Territory communities in Australia between June 2015 and October 2017. Written participant or parent/guardian consent (where participants were aged <18 years) was obtained in all cases, and ethical approval was obtained from the Children’s Health Queensland Human Research Ethics Committee (HREC/14/QRCH/111).

### 2.2. Data Collection

Consenting participants (aged ≥18 years), or parents/guardians (for participants <18 years) completed two self-administered IRRV study questionnaires, with support provided by the research team where required. Information collected included demographic data, respiratory history (including smoking status and household exposure), gestational age at birth, and detailed histories of asthma and atopy. For all participants, paper and electronic medical records from local hospitals and medical centres were used to verify questionnaire data and to collect data on hospitalisations. Where there was disagreement between information collected through participant questionnaires and medical records, information obtained from medical records was used.

### 2.3. Definitions

Ever-pneumonia status was considered verifiable and thus assigned when it was recorded in the participant’s medical records by the treating physician, and the illness was consistent with pneumonia (fever, cough, and respiratory signs or chest radiograph changes). Medical records were also used to assign diagnoses of asthma. Eczema, hay fever, rash, and wheeze were assigned where recorded on study questionnaires.

### 2.4. Spirometry

Spirometry measurements were then performed according to American Thoracic Society (ATS)/European Respiratory Society (ERS) guidelines [22,23] in the seated position with a nose clip, using Easy on-PC spirometers (ndd Medizintechnik, Switzerland) [21]. Z-scores and % predicted values for spirometric measurements were re-calculated for this analysis using ‘Other/mixed’ values from the GLI 2012 reference equations, as these were found be most appropriate for use in First Nations Australians [21].

### 2.5. Participant Selection for the Current Analysis

Children whose spirometry did not meet ATS/ERS criteria were excluded during a previous analysis of the IRRV dataset. [21] For our analysis, we also excluded those with parent-reported pneumonia only (i.e., no verifiable evidence), and children with neonatal pneumonia as we are unable to distinguish between pneumonia and residual pulmonary fluid in foetal lung tissue when reviewing charts retrospectively.

### 2.6. Statistical Analyses

Generalised additive models (GAMs) in R (R Core Team) were used to fit FEV_1_ and FVC Z-scores against age of first pneumonia to test for non-linear associations. Non-linearity was checked using the ‘mgcv’ package with the R software (references provided in supplement). As no compelling evidence for non-linear fits was found, we used univariable linear regression to model FEV_1_ and FVC Z-scores against history of pneumonia (model A) and age of first pneumonia (model B). For the second model, we stratified children into two early childhood groups (less than three years, and between three and five years of age) to explore the effects of pneumonia occurring in different windows within this critical development period, and a third group representing pneumonia not occurring in early childhood (greater than five years). Potential confounders (sex, household smoking, gestational age, eczema, hay fever, rash, and wheeze) were adjusted for in a multivariable linear regression model. Variables with *p* < 0.2 for any level were included in the final models.

Odds ratios (ORs) for asthma occurring later in development were calculated using multivariable logistic regression modelling (adjusted for age, sex, household smoking, gestational age, and wheeze) for children with ever-pneumonia status (model A), and for age of first pneumonia (model B). Stata 16.1 (StataCorp LLC) was used for all other statistical analyses; two-tailed *p*-values < 0.05 were considered significant.

## 3. Results

### 3.1. Study Population

Of 1276 study participants, 355 (28%) were excluded as spirometry did not meet acceptability and/or repeatability criteria [22] or were not undertaken. Eleven participants with parent-recorded but unverifiable pneumonia (where there was no evidence in hospital/clinic charts) were excluded, and one participant with neonatal pneumonia was excluded, leaving 909 participants in our analysis. Their demographic data and spirometry values are summarised in Table 1. A total of 48 subjects had verifiable pneumonia, with 27 subjects first having pneumonia before three years, 13 between three and five years, and 9 after five years of age. Hay fever and wheeze occurred more frequently in subjects with ever-pneumonia status, but household smoking and premature birth appear unrelated to pneumonia status. Participants without spirometry results or whose spirometry did not meet criteria were younger than included participants (median age = 8.1 years, IQR 5.8, 13.0), but other demographic factors were otherwise similar.

### 3.2. Spirometry

Non-linear fitting of FEV_1_ and FVC Z-scores against age of first pneumonia (Supplementary Figures S1 and S2) were not statistically significant in either case (*p =* 0.74; *p =* 0.05 respectively). Univariable and multivariable linear regression modelling of FEV_1_ and FVC Z-scores against exposure of interest was used to explore linear relationships while adjusting for potential confounders and are presented in Table 2 and Table 3, while FEV_1_/FVC% modelling is presented in Supplement Table S3.

**Figure 1:**
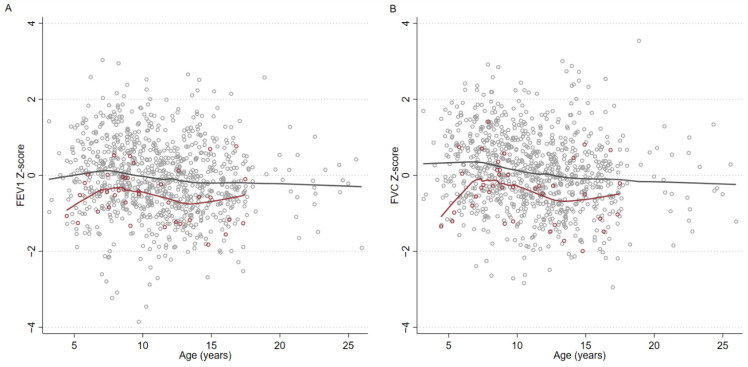
Scatter plots with lowess smoothing comparing FEV_1_ (**A**) and FVC (**B**) Z-scores of First Nations subjects whose pneumonia occurred age ≤5 years versus other subjects. Locally weighted smoothing of participants shows the reduction in lung function occurring post early childhood pneumonia. Gray hollow circles mark participants with no history of pneumonia, or pneumonia occurring after age five years. Gray line is locally weighted smoothing of participants marked with gray hollow circles. Maroon hollow circles mark participants with a history of pneumonia occurring at or before age five years. Maroon line is locally weighted smoothing of participants marked with maroon hollow circles. Age as marked on the horizontal axis indicates the age of the participant when lung function was performed.

Pneumonia was significantly associated with lower FEV_1_ (reduced by 0.35 Z-scores) and FVC (reduced by 0.40 Z-scores). When stratified by age, in those whose pneumonia occurred at before three years of age, FEV_1_ was reduced by 0.42 Z-scores and FVC was reduced by 0.50 Z-scores. When pneumonia occurred at 3–5 years of age, FEV_1_ was reduced by 0.62 Z-scores and FVC was reduced by 0.63 Z-scores. Subjects whose pneumonia occurred age at or before five years were combined and plotted against other subjects (Figure 1), including those whose pneumonia occurred age after five years and those with never-pneumonia status.

There was no significant association between pneumonia status and FEV_1_/FVC% Z-scores including when stratified by age of first pneumonia, and this is evident when subjects whose first pneumonia occurred while aged five years or less were plotted against other subjects (Supplement Figure S4).

### 3.3. Associations with Subsequent Diagnosis of Asthma

Multivariable logistic regression modelling showed ever-pneumonia status was associated with increased risk of developing childhood asthma (OR = 3.10, 95%CI 1.49–6.45). When modelling was repeated with age of first pneumonia as the exposure, we found that pneumonia first occurring before three (OR = 2.67, 95%CI 1.04–6.85) and between three and five years (OR = 8.23, 95%CI 2.73–24.8) of age were associated with an increased risk of childhood asthma. In contrast, pneumonia occurring after five years of age was not associated with increased asthma risk (OR = 2.88, 95%CI 0.38–21.9) but the wide confidence interval suggests a lack of power.

## 4. Discussion

In this study of 909 First Nations Australian children, including 48 children with medical-record-documented pneumonia, we found that pneumonia in early childhood (at or before five years of age) is associated with lower lung function as measured by spirometry in later childhood. There was a reduction in Z-score of >0.4 Z-score across both FEV_1_ and FVC, 95%CI ranging from −1.14 to −0.04 compared to those who never had pneumonia. Pneumonia first occurring after five years of age was not significantly associated with reduced lung function, but this analysis was limited by a small sample size. Overall, associations between age of first pneumonia and spirometry Z-scores appeared to be linear in nature. In addition to pneumonia, other independent predictors of FEV_1_ Z-scores were household smoke exposure, age when spirometry was undertaken, wheeze, and gestational age <30 weeks, with only the last factor having a larger effect size then pneumonia. Other factors impacting on FVC Z-scores were only gestational age <30 weeks and age when spirometry was undertaken. Finally, we demonstrated that pneumonia first occurring between birth and five years of age is also associated with increased risk of childhood asthma, independent of wheeze and other factors such as age, household smoking, and gestational age.

Our study is novel for several reasons. This is the first investigation of the impact of early childhood pneumonia on subsequent child/young adult lung function years later, in a population at high risk of pneumonia and chronic lung disease [4,20]. We demonstrated a significant association between early childhood pneumonia and reduced FEV_1_ and FVC values in later childhood/early adulthood in the First Nations Australian population, independent of age at spirometry testing, household smoking, gestational age, and wheeze. Several studies have found significant reductions in FEV_1_ values in later childhood, [5,6,8,10] however only one other study found a reduction in FVC (−0.25 [95%CI −0.40, −0.10]) [6]. Our data suggest FVC is significantly affected, with an estimated effect size one-half and one-third that of very preterm birth (< 30 weeks) in children less than three and between three and five years of age, respectively. We were unable to demonstrate that pneumonia has any impact on FEV_1_/FVC%, and so the impact to lung function in this cohort of children appears to be restrictive in nature. Previous reports have found obstructive or mixed deficits in children following early childhood pneumonia, with restrictive deficits found in adult populations [5,8,11,12,13]. Secondly, our study’s findings also suggest an association between early childhood pneumonia and development of asthma in later childhood in the First Nations Australian population. It has been demonstrated in other populations that getting pneumonia at an age less than three years old increases the likelihood of physician-diagnosed asthma [9,10]. In the context of the known impact of low lung function in young adulthood on future health [19,20] and the very high morbidity and premature mortality from cardio-respiratory illness in First Nations Australians, we believe that our study’s findings are important.

Our study is exploratory in nature and limited by a small number of children with a history of pneumonia, especially in children aged five years or less. Despite the small sample size and imprecision of our measurements, our effect size estimates are comparatively large compared to previous studies of paediatric populations. Early childhood pneumonia has been previously estimated to reduce childhood FEV_1_ Z-scores by 0.25 (95%CI 0.04, 0.50) [5] and 0.34 (95%CI 0.18, 0.50) [6] respectively, while the only study to find a reduction in FVC estimated the effect to be 0.25 Z-scores (95%CI 0.10–0.40) [5]. When compared to our modelling of pneumonia occurring between birth to age five, we estimate the effects of childhood pneumonia on the lung function of First Nations children to be one to three times larger (based on confidence intervals) than that of other populations studied. These findings suggest that at-risk populations whose incidence of pneumonia is disproportionately high may also experience disproportionate impacts on lung function, and thus related morbidity and mortality. Why this is so can only be speculated, but may include differences in organisms, intrinsic factors such as differential immuno-inflammatory responses to infections, environmental differences, or interactions of multiple factors (e.g., gene–infection, infection–environment, or infection-impaired intrinsic factors). For example, humoral immune responses to vaccines are known to be reduced in Australian First Nation People compared to other Australians, shown for hepatitis B [24] and pneumococcal vaccines [25]. While we lack evidence for this speculation, it has been shown that ‘small lungs’ measured at birth do not predispose infants to acute lower respiratory tract infections (ALRIs)/pneumonia, but does have a multiplicative effect [7]. The Drakenstein child health study prospective cohort study where the infants had their lung function measured at six weeks of age (using multiple breath washout (MBW) and forced oscillation technique) showed conclusively that ALRI impaired lung function when remeasured aged one year, independent of baseline lung function [7].

The clinical significance of reduced lung function is not limited to respiratory disease. Recent studies have shown that reduced lung function, even when still within the clinically normal range, is associated with increased risk of cardiovascular disease and all-cause mortality [19]. These associations have now also been demonstrated in First Nations Australians [20]. In a recent study of 351,874 adults, preserved ratio impaired spirometry, a pattern we observed in participants with a history of early childhood pneumonia, had significantly higher risk of breathlessness, multimorbidity, cardio-vascular disease and increased risk of death, that were unrelated to smoking, obesity, or existing lung disease [26]. These associations are of heightened significance in First Nations Australians, for whom cardiovascular disease is the leading cause of morbidity and mortality (1.2- and 1.6-times higher than other Australians, respectively) [27]. Attaining normal lung function in adulthood is thus important, and the foundations are established in early childhood. There is now evidence that catch-up lung function trajectory improvement is possible in childhood [15,16], and so timely and appropriate management of respiratory disease and infections is critical. We had previously shown that lung function among First Nations Australians can be improved with appropriate clinical management [28]. To achieve this, understanding factors that modify childhood lung function, especially during critical windows of lung development, is important.

Our study has several limitations. Our sample size is small, and we lack the power to explore other associations (e.g., with other respiratory disease). Despite this, our reported effect sizes are larger than previously found in other paediatric populations, and our findings are consistent with current literature regarding lung function impairment post-pneumonia in early childhood. Secondly, our data are based only from First Nations children in Queensland and the Northern Territory and so may limit the generalizability of our findings to the wider First Nations population across Australia. Thirdly, we cannot dismiss the possibility of reverse causation whereby low lung function predisposes children to infection. However, this is an unlikely limitation, as low lung function at six weeks of age (as measured using MBW) was not associated with increased lower respiratory tract infections (LRTI) within the first year of life, but LRTIs were associated with lower lung function measured at 12 months [7]. Finally, 28% of participants either did not have spirometry testing, or their results did not meet acceptability or repeatability criteria. Overall, these children had similar demographic characteristics to those who were included in the analysis except for age, which was included in our modelling as an effect modifier and so is unlikely to affect our findings.

In conclusion, our findings suggest that early childhood pneumonia is associated with lung function deficits in the form of reduced FEV_1_ and FVC values, and development of asthma in later childhood. Given the extremely high prevalence of pneumonia in children, particularly in developing countries and disadvantaged Indigenous populations of affluent countries, it is important to understand its impact on lung development. Indeed, pneumonia incidence among young First Nations Australian children is among the highest reported in the world. In the context of growing evidence of childhood lung function tracking well into adulthood, a better understanding of the impact of pneumonia may inform future research and investment in populations severely affected to improve outcomes through childhood and into adulthood.

## Figures and Tables

**Table 1:** Summary of demographics and lung function at the time of lung function testing for 909 First Nations subjects, including 48 subjects with ever-pneumonia status during critical development windows.

		All Subjects (*n =* 909)	Never-Pneumonia (*n =* 861)	Ever-Pneumonia (*n =* 48)	Pneumonia Occurred When <3 Years of Age (*n =* 26)	Pneumonia Occurred When 3–5 Years of Age (*n =* 13)	First pneumonia When >5 Years of Age (*n =* 9)
		Median (IQR) or *n* (%)					
Age when lung function undertaken		11.0 (8.2, 13.8)	11.0 (8.2, 13.8)	10.1 (8.1, 14.1)	9.1 (8.0, 14.1)	9.1 (7.4, 11.9)	13.4 (11.6, 16.7)
Female		464 (51%)	442 (51%)	22 (46%)	9 (35%)	7 (54%)	6 (67%)
Household smoking		130 (14%)	126 (15%)	4 (8%)	2 (8%)	1 (8%)	1 (11%)
Gestation age (weeks)	>36	464 (51%)	438 (51%)	26 (54%)	15 (58%)	7 (54%)	4 (44%)
	34–36	45 (5%)	42 (5%)	3 (8%)	1 (4%)	1 (8%)	1 (11%)
	30–33	10 (1%)	10 (1%)	0 (0%)	0 (0%)	0 (0%)	0 (0%)
	<30	6 (1%)	6 (1%)	0 (0%)	0 (0%)	0 (0%)	0 (0%)
	Unknown	385 (42%)	365 (42%)	20 (42%)	10 (38%)	5 (38%)	4 (44%)
Eczema		48 (5%)	45 (5%)	3 (6%)	2 (8%)	0 (0%)	1 (11%)
Hayfever		59 (6%)	53 (6%)	6 (12%)	4 (15%)	0 (0%)	0 (0%)
Rash		44 (5%)	43 (5%)	1 (2%)	0 (0%)	0 (0%)	1 (11%)
Wheeze		115 (13%)	104 (12%)	11 (23%)	6 (23%)	1 (8%)	2 (22%)
FEV_1_	Litres (L)	2.02 (1.51, 2.77)	2.03 (1.52, 2.77)	1.97 (1.38, 2.75)	1.77 (1.32, 2.76)	1.74 (1.35, 2.12)	2.73 (2.13, 2.99)
	Z–score	−0.13 (−0.71, 0.54)	−0.11 (−0.70, 0.58)	−0.42 (−1.01, 0.12)	−0.51 (−1.07, −0.04)	−0.44 (−1.17, −0.24)	0.01 (−0.43, 0.42)
	% Predicted	98.5 (91.5, 106.4)	98.7 (91.6, 106.8)	94.9 (87.0, 100.2)	94.0 (86.0, 99.6)	94.7 (85.8, 97.2)	100.1 (95.1, 104.9)
FVC	Litres (L)	2.33 (1.74, 3.18)	2.34 (1.75, 3.18)	2.26 (1.60, 3.22)	1.99 (1.54, 3.39)	1.99 (1.55, 2.38)	3.03 (2.61, 3.44)
	Z–score	0.04 (−0.58, 0.72)	0.06 (−0.58, 0.73)	−0.30 (−1.00, 0.21)	−0.28 (−1.14, 0.14)	−0.47 (−1.03, −0.31)	0.05 (−0.34, 0.81)
	% Predicted	100.4 (93.3, 108.3)	100.6 (93.3, 108.6)	96.6 (88.2, 102.5)	97.0 (87.6, 101.6)	94.7 (88.8, 96.5)	100.5 (96.0, 109.2)

**Table 2:** Univariable and multivariable regression modelling of FEV_1_ Z-scores in 909 First Nations subjects.

		Univariable (*n =* 909)		Multivariable (*n =* 909)			
				Model A		Model B	
		β (95%CI)	*p*	β (95%CI)	*p*	β (95%CI)	*p*
Age when lung function undertaken (per 1 year increase)		−0.03 (−0.05, −0.01)	<0.01	−0.03 (−0.04, −0.01)	<0.01	−0.03 (−0.05, −0.01)	<0.01
Sex (Female) (*n =* 465)		0.01 (−0.12, 0.13)	0.93				
Household smoking (*n =* 130)		−0.19 (−0.37, −0.01)	0.04	−0.19 (−0.37, −0.01)	0.04	−0.19 (−0.37, −0.01)	0.03
Gestational age (weeks)	>36 (*n =* 464)	Reference	-	Reference	-	Reference	-
	34–36 (*n =* 45)	−0.01 (−0.30, 0.29)	0.97	−0.00 (−0.29, 0.29)	1.00	−0.01 (−0.30, 0.29)	0.96
	30–34 (*n =* 10)	−0.46 (−1.07, 0.14)	0.13	−0.40 (−1.01, 0.20)	0.19	−0.40 (−1.00, 0.21)	0.20
	<30 (*n =* 6)	−1.06 (−1.84, −0.27)	<0.01	−0.95 (−1.73, −0.17)	0.02	−0.95 (−1.72, −0.17)	0.02
	Unknown (*n =* 385)	−0.14 (−0.27, −0.01)	0.03	−0.15 (−0.28, −0.02)	0.03	−0.15 (−0.28, −0.01)	0.03
Eczema (*n =* 48)		−0.16 (−0.45, 0.12)	0.26				
Hay fever (*n =* 59)		−0.18 (−0.43, 0.08)	0.18	−0.07 (−0.34, 0.19)	0.58	−0.06 (−0.33, 0.20)	0.64
Rash (*n =* 44)		−0.15 (−0.45, 0.15)	0.32				
Wheeze (*n =* 115)		−0.29 (−0.48, −0.09)	<0.01	−0.22 (−0.42, −0.02)	0.03	−0.23 (−0.43, −0.03)	0.02
Ever-pneumonia (*n =* 49)		−0.34 (−0.62, −0.06)	0.02	−0.35 (−0.63, −0.07)	0.01		
Age (years) of first pneumonia	Never (*n =* 464)	Reference	-			Reference	-
	0–2 (*n =* 27)	−0.40 (−0.78, −0.02)	0.04			−0.42 (−0.79, −0.04)	0.03
	3–5 (*n =* 13)	−0.54 (−1.07, −0.00)	0.05			−0.62 (−1.14, −0.09)	0.02
	>5 (*n =* 9)	0.13 (−0.51, 0.77)	0.70			0.23 (−0.40, 0.86)	0.48

**Table 3:** Univariable and multivariable regression modelling of FVC Z-scores in 909 First Nations subjects.

		Univariable (*n =* 909)		Multivariable (*n =* 909)			
				Model A		Model B	
		β (95%CI)	*p*	β (95%CI)	*p*	β (95%CI)	*p*
Age when lung function undertaken (per 1 year increase)		−0.04 (−0.06, −0.03)	<0.01	−0.04 (−0.06, −0.03)	<0.01	−0.04 (−0.06, −0.03)	<0.01
Sex (Female) (*n =* 465)		0.06 (−0.07, 0.19)	0.33				
Household smoking (*n =* 130)		−0.15 (−0.33, 0.04)	0.12	−0.16 (−0.34, 0.03)	0.09	−0.16 (−0.34, 0.02)	0.09
Gestational age (weeks)	>36 (*n =* 464)	Reference	-	Reference	-	Reference	-
	34–36 (*n =* 45)	0.07 (−0.23, 0.37)	0.66	0.07 (−0.22, 0.37)	0.63	0.06 (−0.23, 0.36)	0.66
	30–34 (*n =* 10)	−0.33 (−0.95, 0.30)	0.30	−0.32 (−0.93, 0.29)	0.30	−0.32 (−0.93, 0.29)	0.30
	<30 (*n =* 6)	−1.23 (−2.03, −0.43)	<0.01	−1.23 (−2.01, −0.44)	<0.01	−1.23 (−2.01, −0.45)	<0.01
	Unknown (*n =* 385)	−0.14 (−0.27, −0.00)	0.05	−0.11 (−0.24, 0.03)	0.12	−0.10 (−0.23, 0.03)	0.14
Eczema		−0.11 (−0.40, 0.18)	0.47				
Hay fever		0.02 (−0.24, 0.28)	0.88				
Rash		−0.13 (−0.43, 0.18)	0.41				
Ever-wheeze (*n =* 115)		−0.04 (−0.24, 0.15)	0.67				
Ever-pneumonia (*n =* 49)		−0.37 (−0.66, −0.09)	0.01	−0.40 (−0.68, −0.12)	<0.01		
Age (years) of first pneumonia	Never (*n =* 464)	Reference	-			Reference	-
	0–2 (*n =* 27)	−0.45 (−0.83, −0.06)	0.02			−0.50 (−0.88, −0.12)	0.01
	3–5 (*n =* 13)	−0.56 (−1.10, −0.01)	0.05			−0.63 (−1.17, −0.10)	0.02
	>5 (*n =* 9)	0.10 (−0.56, 0.75)	0.77			0.22 (−0.42, 0.87)	0.49

## Data Availability

Data collected for this study will not be made publicly available in any form.

## Supplementary Materials

The following are available online at https://www.mdpi.com/article/10.3390/jcm10245727/s1, Supplement Figure S1: Generalised additive modelling of FEV_1_ Z-scores against age of first pneumonia diagnosis. Supplement Figure S2: Generalised additive modelling of FVC Z-scores against age of first pneumonia diagnosis. Supplement Table S3: Univariable and multivariable regression modelling of FEV_1_/FVC Z-scores in 909 First Nations subjects. Supplement Figure S4: Scatter plots with lowess smoothing comparing FEV_1_/FVC% Z-scores of First Nations subjects whose pneumonia occurred age ≤5 years versus other subjects.
