# Supplementary material for: Early Childhood Pneumonia Is Associated with Reduced Lung Function and Asthma in First Nations Australian Children and Young Adults
Source: J Clin Med. 2021 Dec 7;10(24):5727. doi: 10.3390/jcm10245727 (PMC8706766; doi:10.3390/jcm10245727)
Supplement: Supplementary file 1 [file jcm-10-05727-s001.zip › jcm-1484098-supplementary.pdf]

Supplementary Material

Generalised additive modelling using the mgcv[1] package with freeware R.[2]

Supplement Figure S1. Generalised additive modelling of FEV_1 Z-scores against age of first pneumonia diagnosis.

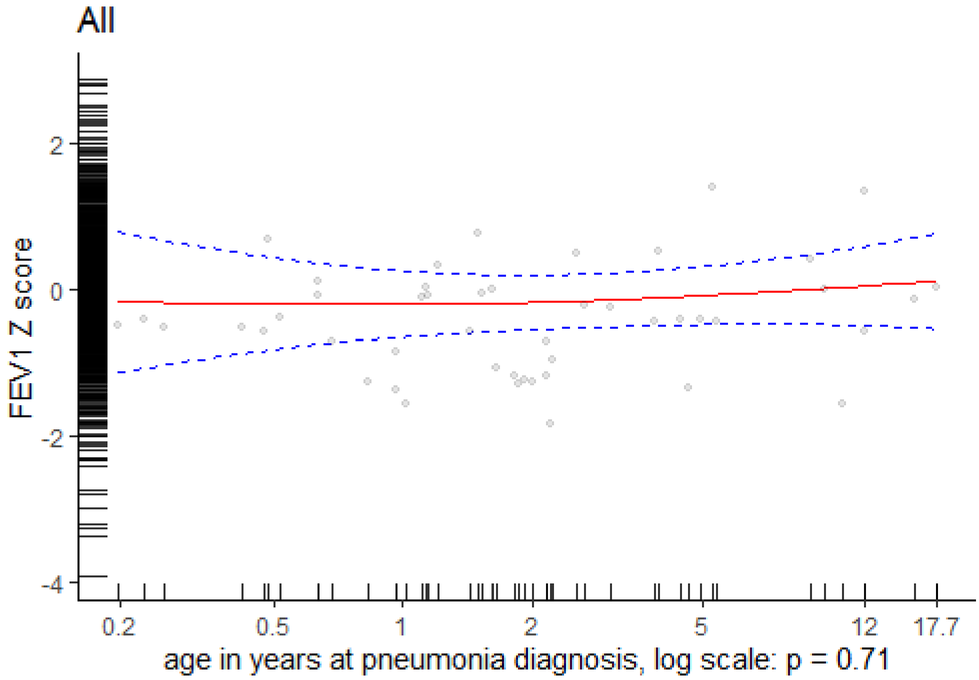

Modelled adjusted for age when lung function undertaken and household smoking.

Supplement Figure S2. Generalised additive modelling of FVC Z-scores against age of first pneumonia diagnosis.

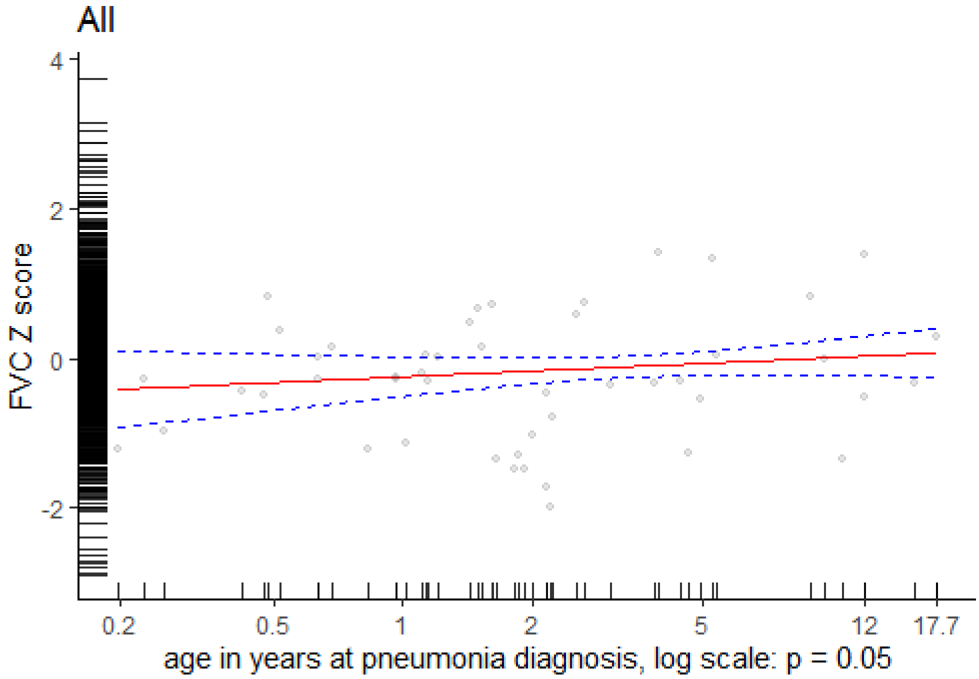

Modelled adjusted for age when lung function undertaken and household smoking.

Supplement Table S3. Univariable and multivariable regression modelling of FEV_1/FVC Z-scores in 909 First Nations subjects

		Univariable (n=909)		Multivariable (n=909)			
				Model 1		Model 2	
		β (95%CI)	p	β (95%CI)	p	β (95%CI)	p
Age when lung function undertaken (per 1 year increase)		0.02 (0.01, 0.04)	<0.01	0.02 (0.01, 0.04)	<0.01	0.02 (0.01, 0.04)	<0.01
Sex (Female) (n=465)		-0.16 (-0.27, -0.04)	<0.01	-0.17 (-0.28, -0.06)	<0.01	-0.16 (-0.28, -0.05)	<0.01
Household smoking (n=130)		-0.09 (-0.26, 0.07)	0.27				
Gestational age (weeks)	>36 (n=464)	Reference	-				
	34-36 (n=45)	-0.15 (-0.42, 0.12)	0.27				
	30-34 (n=10)	-0.31 (-0.87, 0.24)	0.27				
	<30 (n=6)	0.16 (-0.55, 0.87)	0.66				
	Unknown (n=385)	-0.03 (-0.15, 0.09)	0.58				
Eczema		-0.12 (-0.38, 0.14)	0.36				
Hayfever		-0.33 (-0.56, -0.09)	<0.01	-0.22 (-0.46, 0.01)	0.07	-0.23 (-0.47, 0.01)	0.06
Rash		-0.08 (-0.35, 0.19)	0.55				
Ever-wheeze (n=115)		-0.43 (-0.61, -0.26)	<0.01	-0.42 (-0.59, -0.24)	<0.01	-0.42 (-0.59, -0.24)	<0.01
Ever-pneumonia (n=49)		0.02 (-0.24, 0.27)	0.90	0.04 (-0.21, 0.29)	0.76		
Age (years) of first pneumonia	Never (n=464)	Reference	-			Reference	-
	0–2 (n=27)	0.08 (-0.27, 0.42)	0.66			0.13 (-0.21, 0.47)	0.45
	3–5 (n=13)	-0.09 (-0.58, 0.39)	0.71			-0.09 (-0.57, 0.38)	0.70
	>5 (n=9)	-0.00 (-0.59, 0.58)	0.99			-0.03 (-0.61, 0.54)	0.91

Supplement Figure S4. Scatter plots with lowess smoothing comparing FEV_1/FVC% Z-scores of First Nations subjects whose pneumonia occurred age ≤5 years versus other subjects. Gray hollow circle marks participants with no history of pneumonia, or pneumonia occurring after age five years. Gray line is locally weighted smoothing of participants marked with gray hollow circles. Maroon hollow circle marks participants with a history of pneumonia occurring at or before age five years. Maroon line is locally weighted smoothing of participants marked with maroon hollow circles. Age as marked on the horizontal axis indicates the age of the participant when lung function was performed.

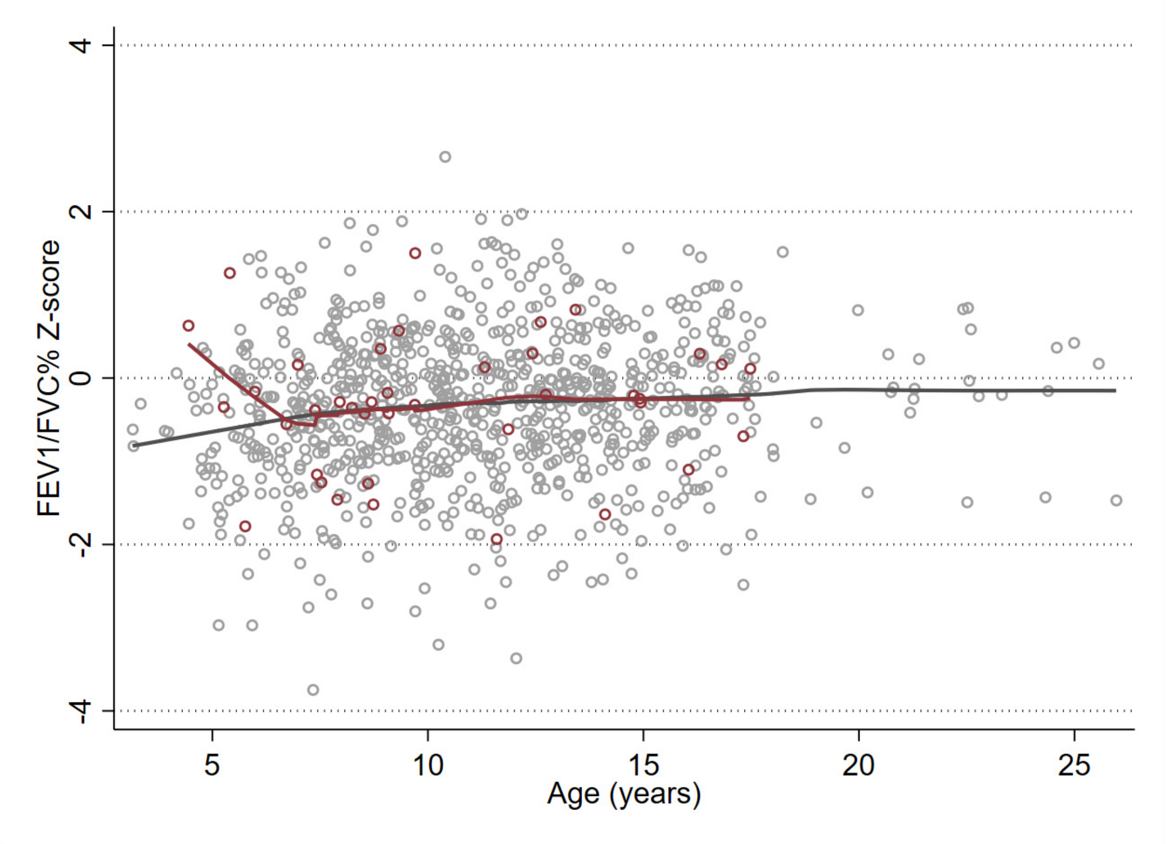

References

1. Wood SN. Generalized Additive Models. 2017.
2. Team RC. R: A language and environment for statistical computing. *MSOR connections*. 2014.
